# The microbial killing capacity of aqueous and gaseous ozone on different surfaces contaminated with dairy cattle manure

**DOI:** 10.1371/journal.pone.0196555

**Published:** 2018-05-14

**Authors:** Ameer Megahed, Brian Aldridge, James Lowe

**Affiliations:** 1 Department of Veterinary Clinical Medicine, College of Veterinary Medicine, University of Illinois at Urbana-Champaign, Urbana, Illinois, United States of America; 2 Department of Animal Medicine, Internal Medicine, Faculty of Veterinary Medicine, Benha University, Moshtohor-Toukh, Kalyobiya, Egypt; Babasaheb Bhimrao Ambedkar University, INDIA

## Abstract

A high reactivity and leaving no harmful residues make ozone an effective disinfectant for farm hygiene and biosecurity. Our objectives were therefore to (1) characterize the killing capacity of aqueous and gaseous ozone at different operational conditions on dairy cattle manure-based pathogens (MBP) contaminated different surfaces (plastic, metal, nylon, rubber, and wood); (2) determine the effect of microbial load on the killing capacity of aqueous ozone. In a crossover design, 14 strips of each material were randomly assigned into 3 groups, treatment (n = 6), positive-control (n = 6), and negative-control (n = 2). The strips were soaked in dairy cattle manure with an inoculum level of 10^7^–10^8^ for 60 minutes. The treatment strips were exposed to aqueous ozone of 2, 4, and 9 ppm and gaseous ozone of 1and 9 ppm for 2, 4, and 8 minutes exposure. 3M™ Petrifilm™ rapid aerobic count plate and plate reader were used for bacterial culture. On smooth surfaces, plastic and metal, aqueous ozone at 4 ppm reduced MBP to a safe level (≥5-log_10_) within 2 minutes (6.1 and 5.1-log_10_, respectively). However, gaseous ozone at 9 ppm for 4 minutes inactivated 3.3-log_10_ of MBP. Aqueous ozone of 9 ppm is sufficient to reduce MBP to a safe level, 6.0 and 5.4- log_10,_ on nylon and rubber surfaces within 2 and 8 minutes, respectively. On complex surfaces, wood, both aqueous and gaseous ozone at up to 9 ppm were unable to reduce MBP to a safe level (3.6 and 0.8-log_10_, respectively). The bacterial load was a strong predictor for reduction in MBP (P<0.0001, R^2^ = 0.72). We conclude that aqueous ozone of 4 and 9 ppm for 2 minutes may provide an efficient method to reduce MBP to a safe level on smooth and moderately rough surfaces, respectively. However, ozone alone may not an adequate means of controlling MBP on complex surfaces.

## Introduction

In the last few decades, there has been a significant increase in the average size of livestock farms as the industries mature and consolidate to seek greater operational efficiency in the face of stiff domestic and international competition [1]. With increasing farm size, there is a corresponding need for effective disinfection tools to minimize the risk of disease transmission within and between animal populations and to lower the chance of zoonotic disease transmission [2]. This is especially true in the case of manure-based pathogens (MBP) which have various survival times that depend fundamentally on the characteristics of the particular organism. As an example, *E*. *coli* O157:H7 can survive in manure for 42 to 49 days at 37°C it extends to 70 days at 5°C, however *Salmonella enterica* serovar *typhimurium* survives for 14 days at 37°C but only extends to 45 days at 5°C [3].

As an example, MBP can cause several hundred diseases in dairy cattle, and more than 150 can be transmitted to humans through direct contact with animals or indirectly through the environment by vectors such as insects or fomites such as dust particles and food products [4–5]. Therefore, reducing the pathogens load in the environment is considered a best management practice to reduce the spread of infection between animals and minimize the introduction of these pathogens into food chains, environment and consequently transmission to humans. In the dairy industry, 5-log_10_ reduction in MBP is suggested as a safe level for farm biosecurity improvement and prevention of infectious diseases [3, 6]. Several detergent and chemical disinfectants can be used effectively in livestock operations, but all the methods are faced with challenges, such as they last for several hours and most of them can be toxic before and after the breakdown in addition to the acquired resistance [7, 8]. Chlorine based sanitizers can be toxic to humans and wildlife because its breakdown produces trihalomethanes and other carcinogenic halo-organic compounds [9]. Quaternary ammonium compounds are no longer capable of controlling of gram-negative bacteria especially *E*. *coli* and *Salmonella* because the acquired resistance [10]. Accordingly, the utilization of other disinfectants with a high killing capacity, that have a short half-life and decompose to nontoxic molecules are an urgent need for the dairy and other livestock industries.

Ozone (O_3_) is among the most powerful oxidants known with oxidative potential of 2.07 volts, nearly twice the oxidizing potential of chlorine [11]. Ozone is an unstable end product of applying strong energy such as radiation, electricity, or heat to oxygen molecules [12]. The half-life of O_3_ in distilled water is 20–30 minutes at 20°C before converting back to oxygen molecule. However, gaseous O_3_ is more stable with half-life of approximately 12 h in atmospheric air [13]. Temperature, pH, and ozone-oxidizable materials are three main factors greatly impact the decomposition rate of O_3_ and its half-life [13–14]. Because of its strong oxidation potential, O_3_ is very effective against bacteria, even with the concentrations as low as 0.01 ppm it is still toxic [15]. Aqueous O_3_ can directly react with the dissolved organic compounds or can generate radical species such as a hydroxyl radical (OH^-^) that have more oxidative potential (2.83 volts) than O_3_ [16]. Ozone destroys bacteria by attacking the glycoproteins and glycolipids in the cell membrane results in rupture of the cell. Moreover, O_3_ attacks the sulfhdryl groups of certain enzymes results in disruption of normal cellular enzymatic activity and loss of function. Ozone also attacks the purine and pyrimidine bases of nucleic acids which results in damage to DNA [15, 17]. The antimicrobial capacity of O_3_ includes not only bacteria, but molds, viruses, and protozoa [18–19]. Therefore, O_3_ has been suggested as a powerful alternative to traditional disinfectants. Ozone has been used extensively for treating municipal drinking water since 1906 [20], and in many commercial food applications [21]. The success of O_3_ in the decontamination of different types of microbes makes gaseous O_3_ an effective way to inactivate the microbial growth in water conditioning systems and in areas that are inaccessible to ultraviolet light [22]. Also, gaseous O_3_ has been recommended as an alternative to reduce microbial populations and increase the shelf life of fruits and vegetable [23].

Several earlier studies reported a significant reduction in individual MBP, such as *E*. *coli*, *Salmonella typhimurium*, *Listeria monocytogenes*, and *Yersinia enterocolitica* after exposure to O_3_ [20, 24–27]. However, few data exist describing the killing capacity of O_3_ on MBP contaminated different surfaces that would be found in livestock operations and food production systems. Accordingly, the primary objective of the present study was to characterize the killing capacity of aqueous and gaseous O_3_ at different operational conditions on different MBP contaminated surfaces (plastic, metal, nylon, rubber, and wood). To our knowledge, no data exists investigating the effect of bacterial load on the rate of bacterial reduction due to exposure to aqueous O_3_. The secondary objective was to use univariate linear regression to determine the effect of microbial load on the killing capacity of aqueous O_3_.

## Materials and methods

### Preparation of materials

Five materials that had varying degrees of surface complexity were used: plastic (smooth or simple), metal (smooth or simple), nylon (intermediate), rubber (intermediate), and wood (rough or complex). These materials have been selected because these are the most common materials used in dairy operations. Additionally, they provide a reasonable range of surface complexity. For each material, fourteen 7.5 X 2.5 cm strips were prepared, hereafter referred to as the substrates. Sample size was determined from the effect size and variation observed in a preliminary, unreported trial. Plastic substrates were prepared from autoclavable polypropylene translucent walled bottles (Thermo Fisher Scientific, Waltham, MA). Metal substrates were prepared from galvanized metal plates (National MFG Inc., Lincoln, NE). Nylon substrates were obtained from a nylon cutting board (Stanton Trading Inc., Farmingdale, NY). Rubber substrates were obtained from rubber boats (Tingley Inc., Piscataway, NJ). Wood substrates were obtained from pine shims (SBC Inc., Saint-Prosper, Canada). Substrates were sterilized in an autoclave three times at 121°C for 20 minutes immediately prior to use.

### Preparation of cattle manure

Approximately 5 g of freshly voided feces was collected using 20 ml sterile economy sample spoon (Sigma Aldrich, St. Louis, MO) into 384 mL Nasco WHIRL-PAK sample bag with puncture proof tabs (Nasco, Fort Atkinson, WI) from a single apparently healthy cow which was at the University of Illinois Dairy Research Farm (UIDRF). A composite 1 g fecal sample was suspended with 9 mL buffered peptone water (BPW) in a 50 mL sterile conical polypropylene tubes equipped with a lid (Thermo Fisher Scientific, Waltham, MA). The time interval between collecting feces and suspending in BPW was always < 30 minutes. The mixture was vigorously vortexed for 1 min followed by serial 10-fold dilutions in BPW. One milliliter from each dilution was spread on 3M™ Petrifilm™ Rapid Aerobic Count Plate (RAC; 3M™ Microbiology, St. Paul, MN) using 3M™ Petrifilm™ spreader (3M™ Microbiology, St. Paul, MN) in order to quantify the bacterial population in the fecal samples. Tryptic soy agar plates (TSA W/ 5% sheep blood agar; Remel, Lenexa, KS, USA) were also used for bacterial identification with MALDI-TOF mass spectrometry. A final volume of 250 ml of diluted feces with an inoculum level ranged from 10^7^ to 10^8^ colony forming unit (cfu)/mL was prepared and placed in a sterile polypropylene pipette tips (1 mL) box with dimensions of 13 X 11.5 X 5.5 cm (Thermo Fisher Scientific, Waltham, MA).

### Identification of bacteria

Blood agar plates with approximately 50 colonies for each fecal sample were sent to Veterinary Diagnostic Laboratory at the University of Illinois Urbana-Champaign for identification of bacteria using MALDI-TOF mass spectrometry. A single colony not more than 24 hours old was smeared directly onto a cleaned MALDI target (MSP 96 polished-steel target; Bruker Daltonik, Bremen, Germany) using clean, sterile toothpicks, then overlaid with 1 μl 70% formic acid to improve the disruption of the cell. The supernatant was allowed to dry at room temperature then overlaid with 1 μl matrix solution (α-cyano-4-hydroxy-cinnamic acid diluted in 50% acetonitrile and 2.5% trifluoroacetic acid) and left to dry at room temperature. The MALDI target was inserted into Bruker Microflex LT MALDI-TOF mass spectrometer operated in the linear mode and equipped with a 337-nm nitrogen laser using FlexControl 3.3 software (Bruker Daltonik, Bremen, Germany). Two hundred forty laser shots were used to generate each spectrum. The mass spectrum with range of 2,000 to 20,000 m/z was collected. Spectra were analyzed with MALDI Biotyper 2.0 software (Bruker Daltonik) at default settings. The automatic analysis generated a peak list for each sample that matched with reference library using an integrated pattern-matching algorithm.

### Ozone generation

For aqueous O_3_, O_3_ was dissolved in water with concentrations from 1 to 10 ppm by using an OOG1X0 O3 generator manufactured by Origin, Inc. (Princeton, NJ, USA). The generator uses ambient air as feed gas and using built in oxygen concentrator to produce up to 10 g of O_3_ per hour with 7.9% concentration (by weight). Ozone was dissolved in water using Orgin, Inc., custom Venturi injector. The ozonated water was maintained in a recirculation system with the volume of 50 L which was designed to maintain a constant dissolved O3 concentration via the adjustment of O_3_ generator output based on dissolved oxygen sensor feedback. Dissolved O_3_ concentration was measured using OSC1X0 dissolved O_3_ meter by Origin, Inc. The water temperature was controlled using aquarium water chiller AquaEuro USA Max Chill 1 HP (AquaCave, Inc., Gurnee, IL). The system allows for the maintenance of concentration with precession of about 0.5 ppm.

For gaseous O3, O_3_ was produced by an AOG001 O_3_ generator with a nominal output of 1 g of O3 per hour (Origin, Inc., Princeton, NJ, USA). The output of generator was tuned down to produce 50 mg of O3 per hour, which allowed to maintain concentration of 1 to 10 ppm in the experimental chamber. The system allowed for the maintenance of constant temperature and humidity of the air in the treatment chamber in the range from room conditions to 100 degrees Fahrenheit and 100% humidity respectively (the system was not capable to reduce either temperature or humidity below ambient).

### Experimental methods of aqueous ozone

The substrates were soaked in the manure mixture for 60 minutes at room temperature (18–21°C) and relative humidity of 55–60%, then removed aseptically and hung on sterile test tube racks using sterile binder clips and toothpicks to dry for 10 minutes. In a crossover design, the 14 substrates of each material were randomly assigned into 3 groups, treatment (n = 6), positive-control (contaminated with cattle feces; n = 6), and negative-control (laboratory blank, inoculated only with sterile water; n = 2). Each substrate was placed aseptically into a Nasco WHIRL-PAK bag. The substrates in the treatment group were then exposed to 20 ml of water containing 2, 4, or 9 ppm of O_3_ for 2, 4, or 8 minutes. This allowed all strips to be completely covered with ozonated water. The temperature of the ozonated water was 13 to 15°C. After filling, the bags were gently shaken by hand for the duration of the exposure period. The positive and negative controls had 20 mL sterilized distilled water (DW) added to the WHIRL-PAK bag for 2, 4, or 8 minutes.

Two milliliters of the water in the bag (WW) were used for culture, 1 mL was spread directly on RAC Petrifilm™ plate. Five, 10 fold (1 mL into 9 ml of BPW) serial dilutions were then created. One milliliter from each dilution was spread on RAC Petrifilm™ plates. All RAC Petrifilm™ plates were incubated at 37°C for 18 to 24 hours. Only, the plates with average 30–300 colonies were used for calculating the bacterial reduction factor (RF). The RAC Petrifilm™ plates were read using an automated counter (3M Petrifilm Plate Reader; 3M™ Microbiology, St. Paul, MN). The results were stored in Excel spreadsheets (Microsoft Corporation, Redmond, WA, USA). The maximum reading of 3M Petrifilm™ reader is “>999,” which corresponds to a minimal bacterial load of 100,000,000 in the case of 1/10^−5^ dilution. All the results were expressed as the number of cfu/mL.

All substrates of treated and control groups were also thoroughly swabbed with a sterile cotton swab. Each swab was washed in 9 ml of BPW. One mL was then serially diluted three times in 9 ml of BPW. One milliliter from each dilution was spread on RAC Petrifilm™ plate. The culture protocol was similar as that described above.

### Experimental methods of gaseous ozone

For treatment groups, the rack with hanging strips of the substrates were transferred to a chamber (65 X 30 X 25) cm with transparent lid connected to the gaseous O_3_ unit and exposed to gaseous O_3_ at concentrations of 1 or 9 ppm for 2, 4, or 8 minutes. Before transferring the strips to chamber the O_3_ generator was turned on until the required concentration was stable in the generation system and the chamber. The relative humidity was stabilized at 95% during the whole experiment. The rack with hanging strips of the control groups were left in a laminar airflow hood (Baker, Sanford, Maine) for the appropriate exposure time. The exposed strips were aseptically placed into Nasco WHIRL-PAK bag and 20 mL BPW was added. The swabbing and culture protocols were similar as that described above for aqueous O_3_.

### Effect of bacterial load

Nylon substrate was selected to investigate the effect of bacterial load on the killing capacity of aqueous O_3_ because the degree of complexity (roughness) of most surfaces used in dairy operations, especially the feeding utensils are close to the degree of nylon surface complexity, even the smooth surface after a certain time from using, it acquires a certain degree of roughness close to degree of nylon surface roughness. Three fecal dilutions were used, 1/10^−2^, 1/10^−4^, 1/10^−5^, in order to get high, medium, and low, levels of bacterial load on the surface. These dilutions were used based on a preliminary, unreported trial. The soaking, washing, and culture protocols were similar as that described above for aqueous O_3_.

### Surface damage

The physical effect of the aqueous and gaseous O_3_ on the materials was also qualitatively monitored thorough visual comparison of the appearance of the test materials of the treatment group against the control group. The material strips were visually inspected for structural damage, surface degradation, discoloration, or other aesthetic impacts.

### Data and statistical analysis

The log_10_ density for each material strip was calculated using the formula presented in ASTM method E2871-12 [28], as follows:

$${log}_{10}\left(\frac{cfu}{mL}\right)={log}_{10}\left\{\left(\frac{cfu}{volume\;plated}\right)\;X\;(\frac{washing\;solution\;volume}{dilution})\right\}$$

Log_10_ RF and kill percentage (% kill) was calculated by using an equation presented in ASTM method E2871-12 [28], as follows:

$${log}_{10}RF={log}_{10}\;control-{log}_{10}\;treated$$

$$\%kill=(1-10^{-RF})\;X\;100$$

Non-normal data, based on a p<0.05 for the Shapiro-Wilk Statistic or if P<0.05 was obtained from Levene's test, were expressed as median and range. Normal distributed data was expressed as a mean ± SD. Mann-Whitney U-tests or Kruskal-Wallis One Way Analysis of Variance on Ranks were used for comparisons between groups with nonnormal distribution or unequal variances data. For post-hoc comparisons, p-values were adjusted for multiple comparisons according to Tukey. Repeated measures ANOVA was used to detect differences in log_10_ RF between O_3_ concentrations, exposure times, and the interaction between O3 concentrations, exposure times using PROC MIXED (SAS 9.3, SAS Inc., Cary, NC). Whenever the F-test was significant, Bonferroni-adjusted P-values were used to assess differences between different concentrations at a specific time of exposure, and between times of exposure within a specific O_3_ concentration.

Multivariable linear regression (PROC REG) was used to test the effect of the O_3_ concentrations and exposure times, and the interaction between O_3_ concentrations and exposure times on log_10_ RF. The interaction term was dropped from the analysis if O_3_ concentration and the interaction between O_3_ concentrations and exposure times were not significant. Univariate linear regression (PROC REG) was used to evaluate the impact of microbial load on the killing capacity of aqueous O_3_.

Logistic regression (PROC LOGISTIC) was used to characterize the relationship between 5-log_10_ RF as a safe level of MBP [3], was achieved by exposure to aqueous O_3_ of 4 ppm for 4 minutes (1 = RF ≥ 5 cfu/mL; 0 = RF < 5 cfu/mL) and materials. Statistical analyses were performed using SAS 9.3 software (SAS Inc, Cary NC) and Excel spreadsheet (Microsoft Corporation, Redmond, WA, USA).

## Results

The bacteria identified by direct MALDI-TOF mass spectrometry were *Bacillus cereus*, *Citrobacter freundii*, *Enterococcus italicus*, *Escheichia coli*, and *Proteus hauseri*. All associated laboratory blanks were 0 cfu.

### Plastic

**Fig 1:**
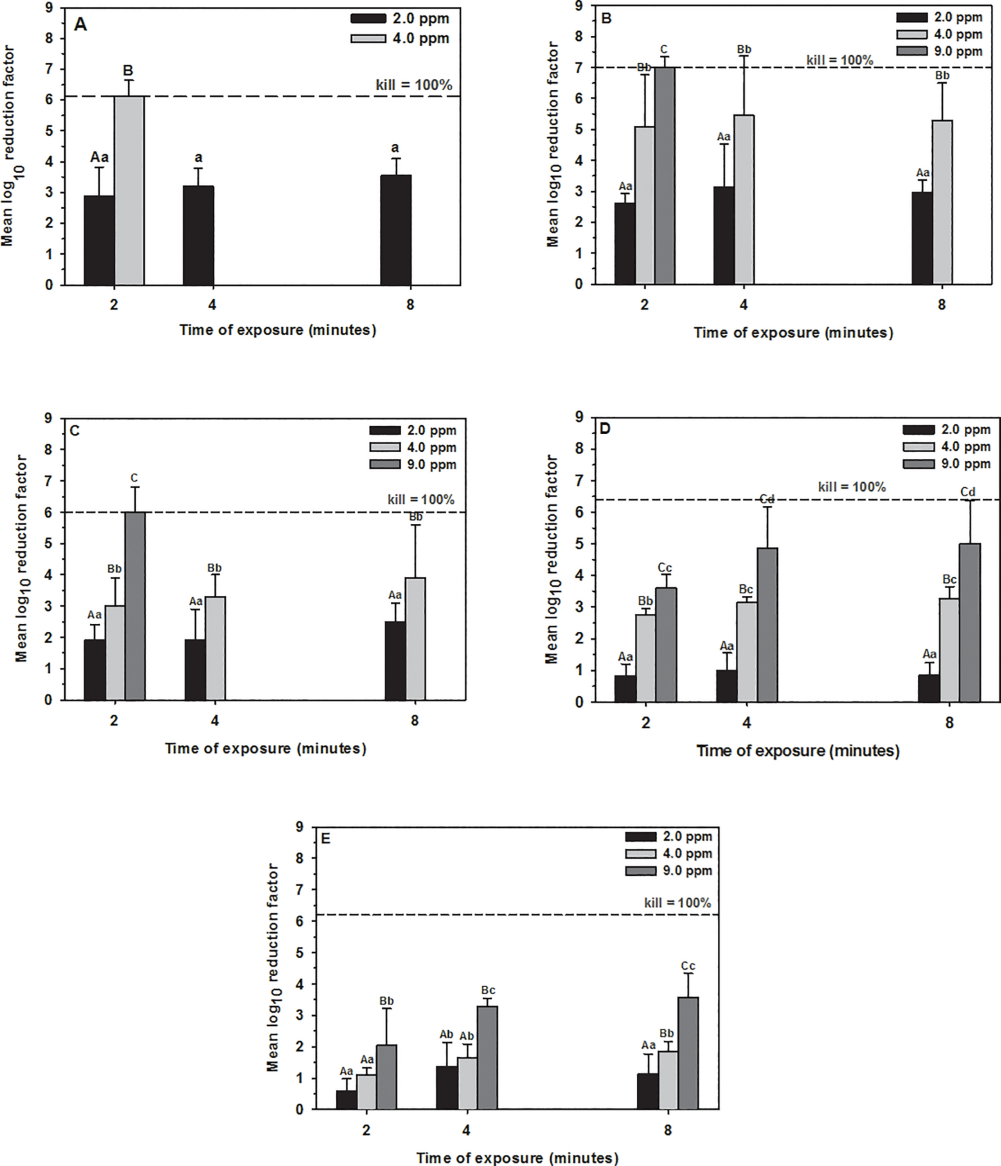
Mean ± SD log_10_ reduction in MBP cell counts in washing water. (A) Plastic, (B) metal, (C) nylon, (D) rubber, and (E) wood substrates contaminated with dairy cattle manure and treated with aqueous O_3_ of 2, 4, and 9 ppm for 2, 4, and 8 minutes exposure. The horizontal dashed line indicates 100 killing percentage (mean cell counts of the control groups at the same concentration and time point). Concentrations at the same time point with different capital letters differ significantly (*P* < 0.05). Time points with different small letters within one concentration differ significantly (P < 0.05).

**Fig 2:**
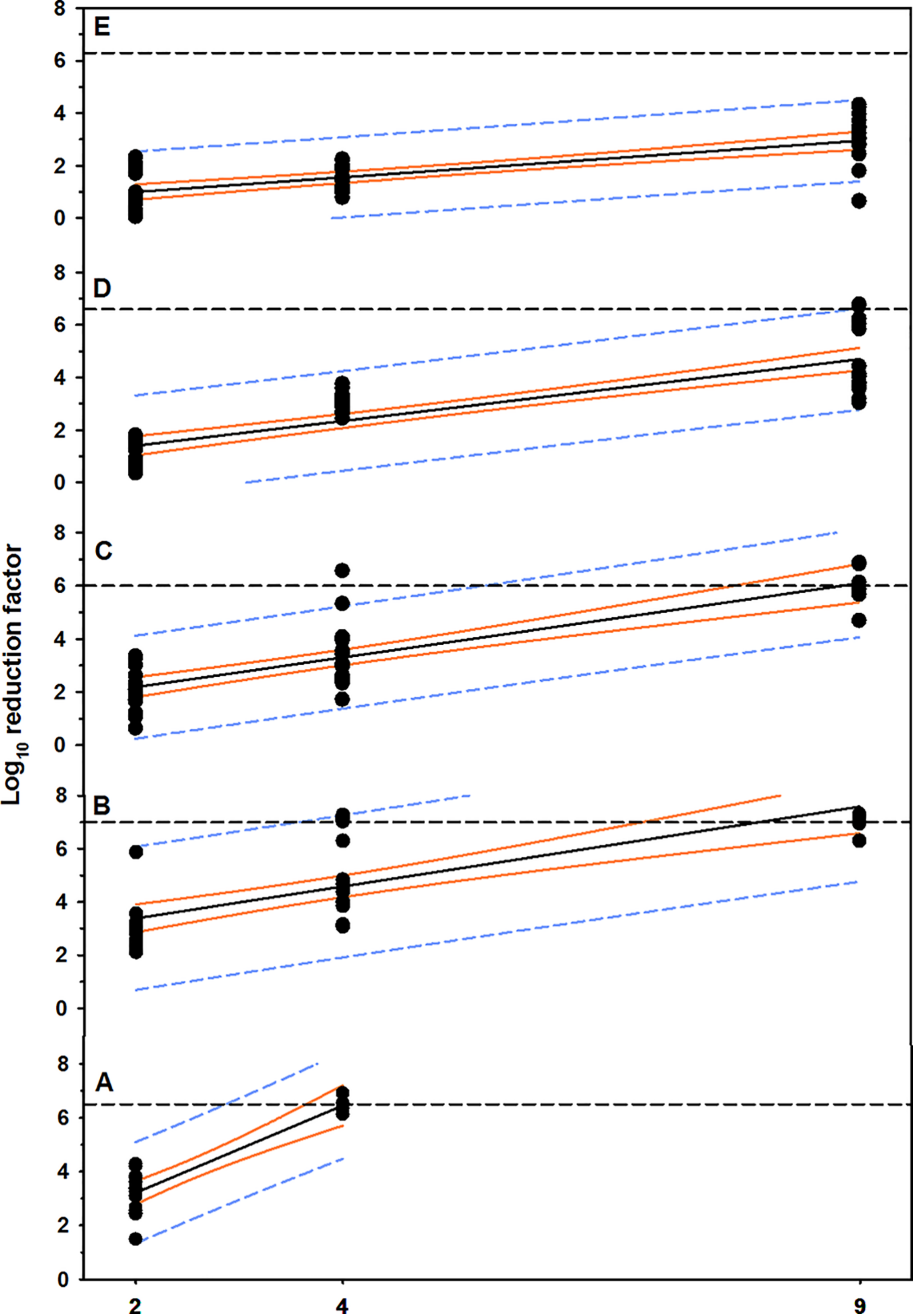
Positive linear relationship between concentrations of aqueous O_3_ and rate of MBP reduction. Plastic, (B) metal, (C) nylon, (D) rubber, and (E) wood materials contaminated with dairy cattle manure. The solid black line is the regression line, the solid orange line is the 95% confidence interval for the regression line, and the short dashed line is the 95% confidence interval for prediction. The horizontal short dashed line is 100 killing percentage (mean cell counts of the control groups).

Aqueous O_3_ at concentration of 4 ppm or greater reduced bacterial load below detectable limits within 2 minutes exposure (Table 1, Fig 1A, S1A Fig). The results of multivariable regression analysis indicated that the reduction in MBP cell counts was dependent on the concentration of aqueous O_3_ (*P* < 0.0001; R^*2*^ = 0.87), while the exposure time has no effect (*P* < 0.0861; Table 2, Fig 2A). However, the mean log_10_ reduction in cell counts increased (P = 0.098) at 4 minutes exposure (3.6 ± 1.1; mean ± SD), compared to 2 minutes exposure (2.9 ± 0.9; Fig 1A).

**Table 1:** Median and range for log_10_ MBP cell counts (cfu/mL) in washing water of control and treated plastic, metal, nylon, rubber, and wood substrates with aqueous O_3_ of 2, 4, and 9 ppm for 2, 4, 8 minutes.

Dose	Exposure time					
	2 min		4 min		8 min	
	Control	O_3_	Control	O_3_	Control	O_3_
**Plastic**						
2.0 ppm	6.1 (5.5,6.8)^a^	3.4 (2.0,4.0)^*^^a^	5.9 (5.5,6.4)	2.8 (0.0,3.1)^*^	6.6 (5.7,6.9)	3.1 (2.3,3.5)^*^
4.0 ppm	6.4 (6.1,6.9)^a^	0.0^*^^b^				
**Metal**						
2.0 ppm	7.1 (7.1,7.3)^a^	4.6 (4.1,4.9)^*^^a^	5.9 (5.4,6.1)^a^	3.1 (0.0,3.7)^*^^a^	6.8 (5.6,6.8)^a^	3.4 (3.1,3.9)^*^^a^
4.0 ppm	7.1 (6.9,7.2)^a^	2.4 (0,4.1)^*^^b^	7.2 (6.3,7.3)^b^	1.6 (0,3.2)^*^^b^	7.1 (6.3,7.3)^a^	2.4 (0,2.7)^*^^b^
9.0 ppm	7.1 (6.3,7.3)^a^	0.0^*^^c^				
**Nylon**						
2.0 ppm	6.0 (5.7,6.7)^a^	4.3 (3.6,4.8)^*^^a^	5.6 (5.4,6.2)^a^	3.7 (2.9,4.8)^*^^a^	5.6 (5.2,6.7)^a^	3.2 (2.8,3.8)^*^
4.0 ppm	5.7 (5.0,6.7)^a^	2.8 (1.9,3.3)^*^^b^	5.4 (5.1,6.0)^a^	2.6 (1.3,2.9)^*^^b^	5.8 (4.8,6.6)^a^	2.6 (0.0,3.3)^*^
9.0 ppm	6.0 (4.7,6.9)^a^	0.0^*^^c^				
**Rubber**						
2.0 ppm	6.0 (5.9,6.2)^a^	5.2 (4.9,5.6)^*^^a^	6.1 (5.6,6.8)^a^	5.0 (4.9,5.8)^*^^a^	6.6 (6.2,6.7)^a^	5.8 (5.1,6.0)^*^^a^
4.0 ppm	6.8 (6.5,6.9)^a^	4.1 (3.6,4.3)^*^^b^	6.7 (6.1,6.8)^a^	3.5 (3.2,3.7)^*^^b^	6.7 (6.3,6.9)^a^	3.5 (3.1,3.6)^*^^b^
9.0 ppm	6.6 (6.4,6.8)^a^	3.0 (2.6,3.6)^*^^c^	6.7 (6.2,6.9)^a^	2.5 (0.0,3.0)^*^^c^	6.6 (5.9,6.8)^a^	1.3 (0.0,3.0)^*^^c^
**Wood**						
2.0 ppm	6.3 (5.9,6.4)^a^	5.6 (4.9,6.3)^*^^a^	6.1 (5.5,7.0)^a^	5.0 (3.9,5.3)^*^^a^	6.4 (5.6,6.8)^a^	5.1 (4.7,5.7)^*^^a^
4.0 ppm	7.1 (6.8,7.3)^b^	6.0 (5.7,6.2)^*^^a^	7.0 (6.3,7.3)^b^	5.3 (4.6,6.0)^*^^a^	7.2 (6.3,7.3)^b^	5.2 (4.9,5.8)^*^^a^
9.0 ppm	5.9 (4.3,6.6)^a^	3.6 (3.1,3.8)^*^^c^	6.7 (6.6,6.8)^a^^b^	3.5 (2.8,3.7)^*^^b^	6.8 (6.2,6.9)^a^^b^	3.0 (2.4,3.8)^*^^b^

*Values within a row are significantly different between control and treated groups for each time point.

^a-c^Values with different letters within a column for each material are significantly different.

**Table 2:** Multiple linear regression model for predicting RF on plastic, metal, nylon, rubber, and wood substrates loaded with MBP and treated with aqueous O_3_ of 2, 4, and 9 ppm and gaseous O_3_ of 1, and 9 ppm for 2, 4, and 8 minutes exposure.

	Coefficient	Estimated value	SE	Probability	Model R^2^
**Plastic**					
**Aqueous**	Intercept	-0.80	0.58	0.1781	0.87
	Concentration	1.76	0.17	<0.0001	
	Exposure time	0.11	0.06	0.0861	
**Gaseous**	Intercept	0.58	0.22	0.0119	0.85
	Concentration	0.30	0.022	<0.0001	
	Exposure time	-0.02	0.04	0.5118	
**Metal**					
**Aqueous**	Intercept	1.74	0.63	0.0088	0.60
	Concentration	0.63	0.09	<0.0001	
	Exposure time	0.08	0.09	0.3801	
**Gaseous**	Intercept	-0.33	0.18	0.0736	0.75
	Concentration	0.16	0.02	<0.0001	
	Exposure time	0.04	0.03	0.1347	
**Nylon**					
**Aqueous**	Intercept	0.32	0.44	0.2047	0.70
	Concentration	0.61	0.06	<0.0001	
	Exposure time	0.13	0.06	0.0571	
**Gaseous**	Intercept	0.10	0.20	0.6233	0.63
	Concentration	0.16	0.02	<0.0001	
	Exposure time	-0.01	0.03	0.8791	
**Rubber**					
**Aqueous**	Intercept	0.02	0.34	0.9426	0.70
	Concentration	0.47	0.04	<0.0001	
	Exposure time	0.09	0.05	0.0412	
**Gaseous**	Intercept	0.58	0.22	0.0119	0.85
	Concentration	0.30	0.02	<0.0001	
	Exposure time	-0.02	0.04	0.5118	
**Wood**					
**Aqueous**	Intercept	-0.20	0.25	0.4269	0.65
	Concentration	0.28	0.03	<0.0001	
	Exposure time	0.14	0.03	0.0005	
**Gaseous**	Intercept	-0.02	0.15	0.8809	0.51
	Concentration	0.09	0.02	<0.0001	
	Exposure time	-0.00	0.03	0.9745	

**Fig 3:**
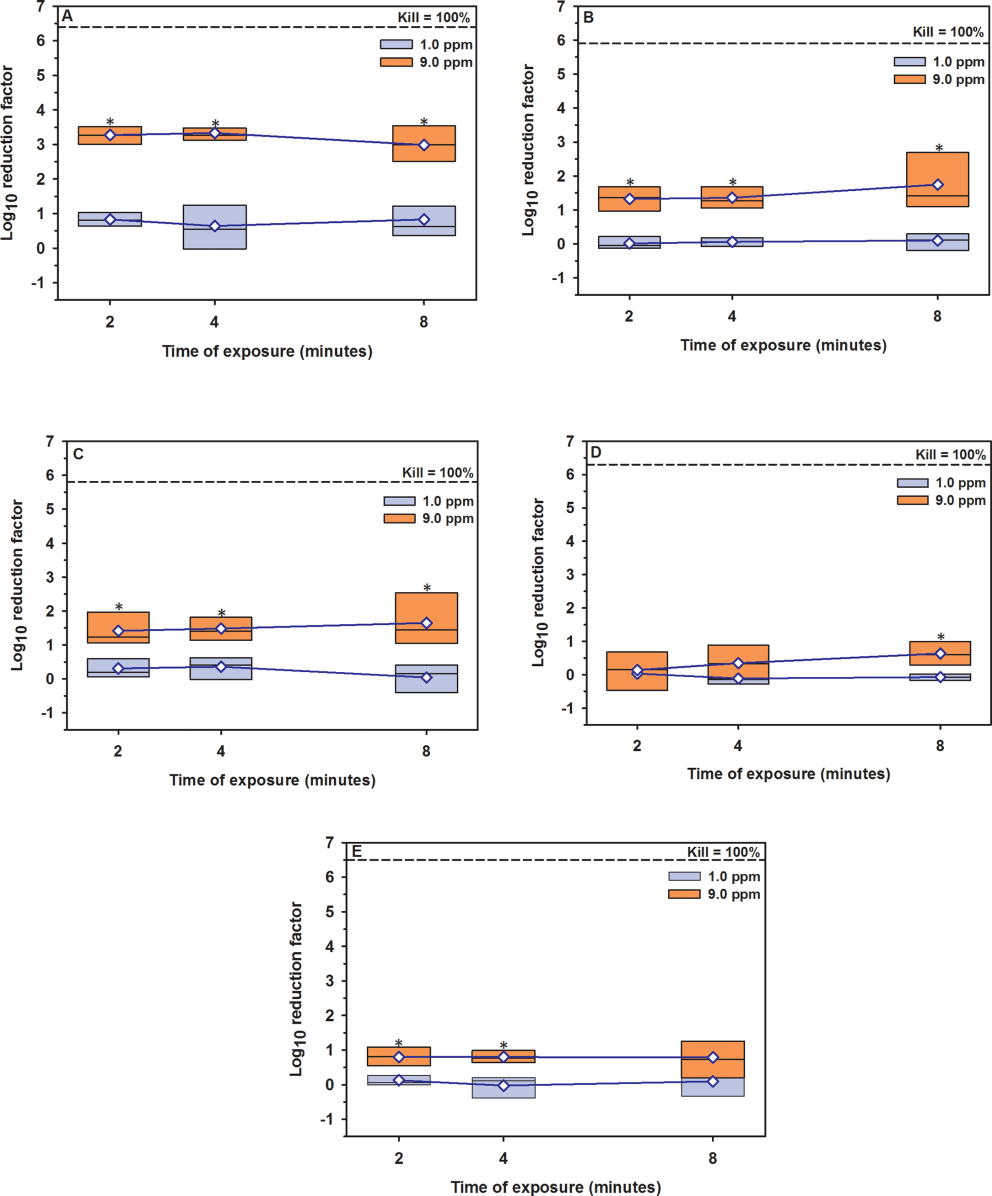
Boxplot of log_10_ reduction in MBP cell counts in washing water. (A) Plastic, (B) metal, (C) nylon, (D) rubber, and (E) wood materials contaminated with dairy cattle manure and treated with gaseous O3 of 1 and 9 ppm for 2, 4, 8 minutes exposure. The horizontal dashed line indicates 100 killing percentage (mean cell counts of the control groups). The blue diamond indicates mean. *Values differ significantly between O_3_ concentrations at the same time point (P < 0.05).

**Fig 4:**
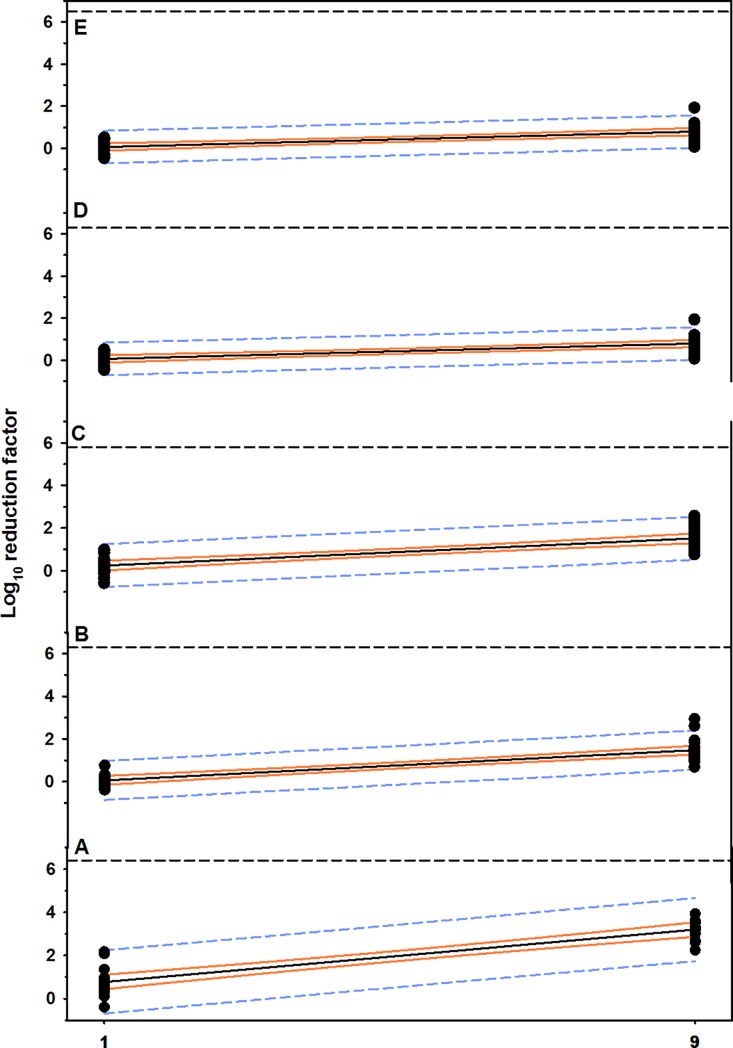
Scatterplot of the linear relationship between concentrations of gaseous O_3_ and rate of MBP reduction. (A) Plastic, (B) metal, (C) nylon, (D) rubber, and (E) wood materials contaminated with dairy cattle manure. The solid black line is the regression line, the solid orange line is the 95% confidence interval for the regression line, and the short dashed line is the 95% confidence interval for prediction. The horizontal short dashed line is 100 killing percentage (mean cell counts of control groups).

On the other hand, gaseous O_3_ at concentration of 9 ppm for 4 minutes killed approximately 3.3-log_10_ of MBP in washing water and 2.0-log_10_ on surface, compared to 0.6 and 0.4-log_10_ RF, respectively, when concentration of 1 ppm for the same exposure time was used (Table 3, Fig 3A, S2A Fig). The results of multivariable regression analysis showed that the concentration of gaseous O_3_ was a significant predictor (*P* < 0.0001) and explained approximately 85% of the reduction in cell counts, however the time of exposure showed no effect (*P* < 0.5118; Table 2, Fig 4A).

**Table 3:** Median and range for log_10_ MBP cell counts (cfu/mL) in washing water of control and treated plastic, metal, nylon, rubber, and wood substrates with gaseous O_3_ of 1 and 9 ppm for 2, 4, 8 minutes.

Exposure time								
	2 min		4 min				8 min	
	Control	O_3_	Control		O_3_		Control	O_3_
**Plastic**								
1.0 ppm	6.4 (6.3,6.6)^a^	5.7 (5.2,5.9)^*^^a^	6.3 (5.7,6.7)^a^		5.9 (4.6,6.2)^a^		6.3 (6.0,6.8)^a^	5.6 (4.6,5.8)^*^^a^
9.0 ppm	6.4 (6.0,6.7)^a^	3.1 (2.8,3.5)^*^^b^	6.5 (6.2,6.6)^a^		3.1 (2.6,3.4)^*^^b^		6.3 (5.7,6.8)^a^	3.1 (2.8,4.2)^*^^b^
**Metal**								
1.0 ppm	6.8 (6.6,6.9)^a^	6.7 (6.5,6.9)^a^	6.7 (6.5,6.9)^a^		6.6 (6.5,6.8)^a^		6.5 (6.3,6.7)^a^	6.5 (5.9,6.7)^a^
9.0 ppm	6.2 (5.9,6.7)^a^	5.0 (4.5,5.3)^*^^b^	5.9 (4.5,6.3)^b^		4.5 (4.3,5.1)^*^^b^		5.7 (3.9,6.9)^b^	4.1 (2.6,4.4)^*^^b^
**Nylon**								
1.0 ppm	6.2 (5.9,6.6)^a^	6.0 (5.5,6.2)^a^	6.2 (5.9,6.5)^a^		5.9 (5.6,6.1)^a^		5.9 (5.5,6.3)^a^	5.8 (5.6,6.1)^a^
9.0 ppm	5.9 (5.7,6.9)^a^	4.8 (3.8,5.1)^*^^b^	5.5 (5.2,6.2)^b^		4.1 (3.6,4.6)^*^^b^		4.5 (4.2,6.2)^b^	3.2 (2.0,3.7)^*^^b^
**Rubber**								
1.0 ppm	6.5 (6.3,6.6)^a^	6.4 (6.3,6.5)^a^	6.3 (6.3,6.6)^a^		6.5 (6.3,6.7)^a^		6.4 (6.3,6.5)^a^	6.4 (6.4,6.6)^a^
9.0 ppm	5.9 (5.5,6.6)^b^	5.7 (5.2,6.5)^b^	5.9 (5.5,6.4)^b^		5.8 (4.5,6.5)^b^		6.4 (6.2,6.7)^a^	5.8 (5.2,6.3)^*^^b^
**Wood**								
1.0 ppm	6.6 (6.5,6.8)^a^	6.5 (6.2,6.7)^a^	6.5 (6.1,6.7)^a^		6.5 (6.3,6.6)^a^		6.2 (6.0,6.7)^a^	6.3 (5.7,6.6)^a^
9.0 ppm	6.7 (6.5,6.8)^a^	5.9 (5.5,6.2)^*^^b^	6.5 (6.5,6.7)^a^		5.8 (5.5,6.1)^*^^b^		6.4 (6.1,6.7)^a^	5.7 (4.8,6.3)^*^^a^

*Values within a row are significantly different between control and treated groups for each time point.

^a-b^Values with different letters within a column for each material are significantly different.

### Metal

Aqueous O_3_ at concentration of 4 ppm reduced MBP in washing water to a safe level within 2 minutes and below detectable limits on surface within 4 minutes; while, exposure to concentration of 9 ppm for 2 minutes reduced the bacterial load below detectable limits (Table 1, Fig 1B, S1B Fig). The results of multivariable regression analysis indicated that the reduction in MBP cell counts was dependent on the concentration of aqueous O_3_ (*P* < 0.0001; R^*2*^ = 0.60) with no effect for the time of exposure (*P* < 0.3801; Table 2, Fig 2B). However, the mean log_10_ RF increased numerically at 4 minutes exposure for 2 and 4 ppm (3.1 ± 1.4, 5.4 ± 1.9), compared to 2 minutes exposure (2.6 ± 0.3, 5.1 ± 1.7, respectively; Fig 1B).

Gaseous O_3_ at concentration of 9 ppm for 8 minutes exposure killed 1.7-log_10_ of MBP in washing water and 1.6-log_10_ on surface (Table 3, Fig 3B, S2B Fig). According to multivariable regression analysis, the concentration of gaseous O_3_ is considered the most powerful predictor (*P* < 0.0001) and explained approximately 75% of the reduction in cell counts, compared to time of exposure (*P* < 0.1347; Table 2, Fig 4B).

### Nylon

Aqueous O_3_ at concentration of 9 ppm or greater reduced MBP in washing water and on surface below detectable limits within 2 minutes exposure (Table 1, Fig 1C, S1C Fig). According to multivariable regression analysis, the concentration of aqueous O_3_ is considered the most powerful predictor (*P* < 0.0001) and explained approximately 70% of the reduction in cell counts, compared to time of exposure (*P* < 0.0571; Table 2, Fig 2C). However, the mean log_10_ reduction in cell counts increased numerically at 4 minutes exposure for 4.0 ppm (3.3 ± 0.7), compared to 2 minutes exposure (2.7 ± 0.5, Fig 1C).

Gaseous O_3_ at concentration of 9 ppm for 4 minutes exposure killed approximately 1.6-log_10_ of MBP in washing water and 1.4-log_10_ on surface (Table 3, Fig 3C, S2C Fig). According to multivariable regression analysis, concentration of gaseous O_3_ is the main predictor (*P* < 0.0001) and explained approximately 63% of the reduction in cell counts, compared to time of exposure (*P* < 0.8791; Table 2, Fig 4C).

### Rubber

Aqueous O_3_ at concentration of 9 ppm reduced 5.0-log_10_ of MBP in washing water after 8 minutes exposure (Table 1, Fig 1D). However, aqueous O_3_ at concentration of 9 ppm cleared the rubber surface from MBP within 2 minutes (S1D Fig). The results of multivariable regression analysis indicated that the reduction in cell counts was dependent on the concentration of aqueous O_3_ (*P* < 0.0001) and time of exposure (*P* < 0.0412) and the final model explained 70% of the reduction in cell counts (Table 2, Fig 2D). The RF of 9 ppm aqueous O_3_ significantly increased at 4 minutes exposure (4.9 ± 1.3, *P* = 0.049), compared to 2 minutes exposure (3.6 ± 0.4; Fig 1D).

Gaseous O_3_ did not result in a significant reduction in MBP cell counts except at a concentration of 9.0 ppm for 8 minutes exposure with average log_10_ reduction of 0.6 in washing water and 1.0 on surface (Table 3, Fig 3D, S2D Fig). According to multivariable regression analysis, the reduction in cell counts was dependent on the concentration of gaseous O3 (*P* < 0.0001) and explained approximately 85% of the reduction in cell counts, while the time of exposure did not show any effect (*P* < 0.5118; Table 2, Fig 4D).

### Wood

Aqueous O_3_ at concentration of 9.0 ppm was unable to reduce MBP below detectable limits and reduced MBP in washing water with average log_10_ reduction of 3.6 at 8 minutes exposure and on surface 1.7-log_10_ (Table 1, Fig 1E, S1E Fig). The results of multivariable regression analysis indicated that the reduction in cell counts was dependent on the concentration of aqueous O_3_ (*P* < 0.0001) and time of exposure (*P* < 0.0005). The final model explained 65% of the reduction in cell counts (Table 2, Fig 2E). Mean log_10_ reduction in cell counts of 2, and 9 ppm aqueous O_3_ significantly increased at 4 minutes exposure (1.4 ± 0.8, *P* = 0.034; 3.3 ± 0.3, *P* = 0.001; respectively), compared to 2 minutes exposure (0.6 ± 0.4, 2.0 ± 1.2, respectively, Fig 1E).

The highest reduction in the MBP cell counts of gaseous O_3_ was at concentration of 9 ppm for 4 minutes exposure with average log_10_ reduction of 0.8 in washing water and 0.9 on surface (± 0.3; Table 3, Fig 3E, S2E Fig). According to multivariable regression analysis, the reduction in cell counts was dependent on the concentration of gaseous O3 (*P* < 0.0001) and explained approximately 51% of the reduction in cell counts, however the time of exposure has no effect (*P* < 0.9745; Table 2, Fig 4E).

### Effect of substrates

**Fig 5:**
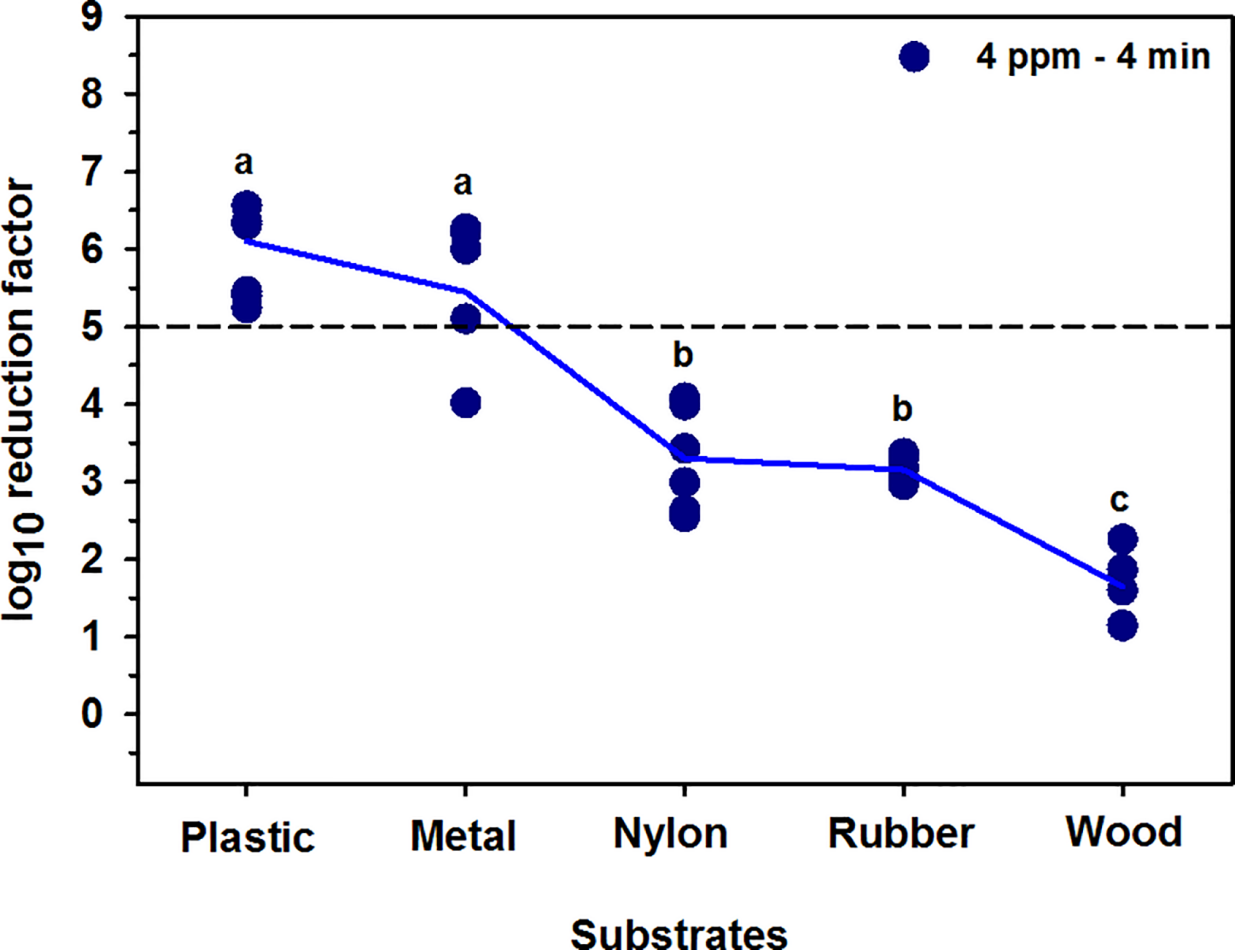
Mean log_10_ reduction in MBP cell counts on plastic, metal, nylon, rubber, and wood. The short horizontal dashed line indicates the safe level of bacterial reduction (>5-log_10_). a-c Values with different letters are significantly different (P < 0.05).

The type of surface has a significant (P < 0.0001) impact on the reduction in MBP cell counts. The averages log_10_ reduction of 4 ppm aqueous O_3_ for 4 minutes exposure for plastic, metal, nylon, rubber, and wood materials were 5.9 (± 0.6), 5.4 (± 1.9), 3.3 (± 0.7), 3.2 (± 0.2), and 1.6 (± 0.4), respectively (Fig 5).

**Fig 6:**
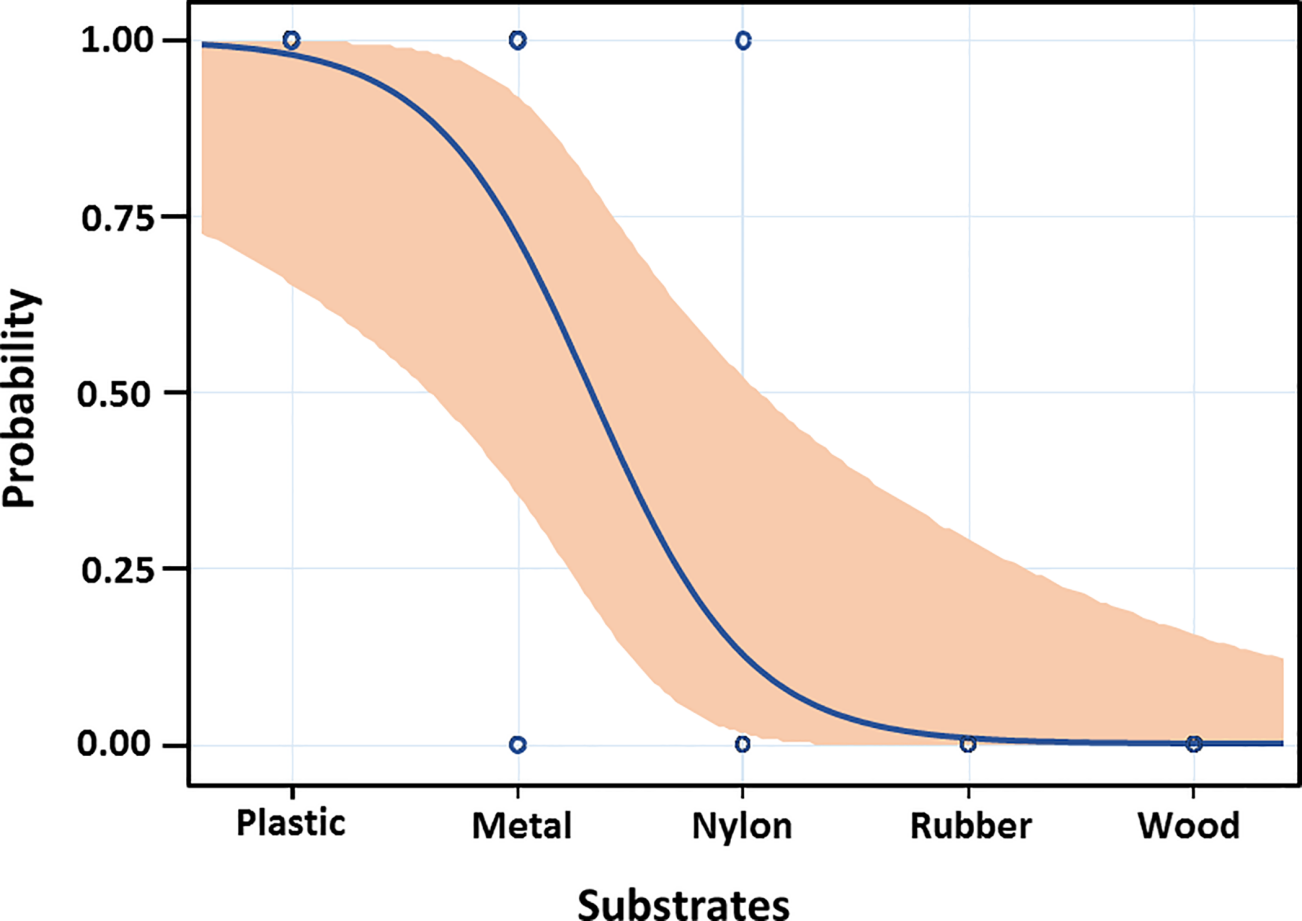
Probability plot for the ability of aqueous O_3_ of 4 ppm for 4 minutes exposure time to decrease the bacterial load with 5-log_10_ on plastic, metal, nylon, rubber, and wood materials. The curve shows the likely probability of 5-log_10_ bacterial reduction for each material, with the 95% confidence interval for the probability.

The likely probability of 5-log_10_ reduction for each material, with the 95% confidence interval for the probability is presented in Fig 6.

### Effect of bacterial load

**Fig 7:**
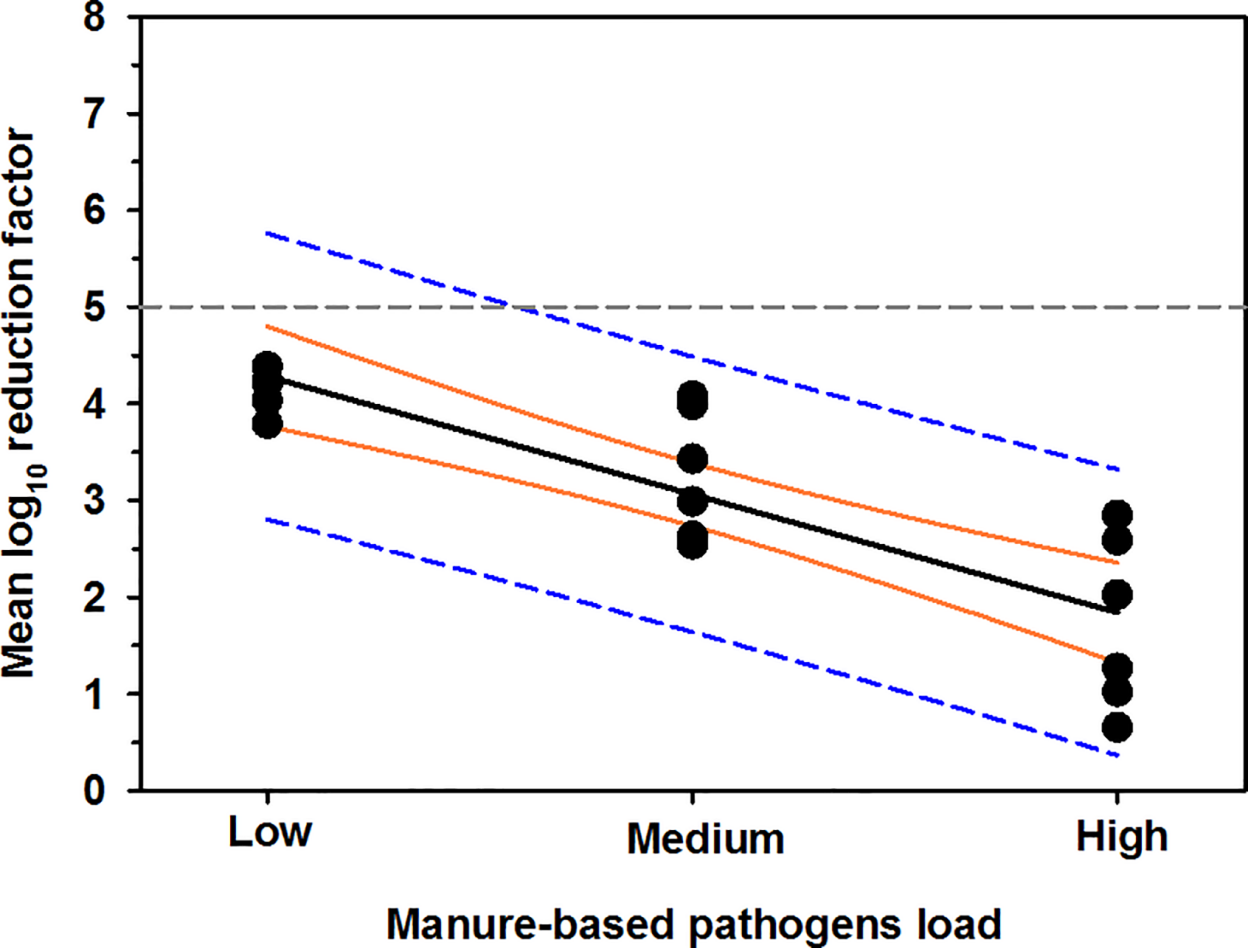
Negative linear relationship between the bacterial load and rate of bacterial reduction on nylon surface. The horizontal gray dotted line represent the safe level of MBP (>5-log_10_). The thick solid black line is the regression line, the orange lines are the 95% confidence interval for regression line, and the blue dashed lines are the 95% confidence interval for prediction.

The averages log_10_ cell counts of control groups of low, medium, and high MBP load on nylon surface were 4.2 (± 0.2), 5.5 (± 0.3), and 8.4 (± 0.8), respectively. The averages log_10_ RF of low, medium, and high MBP load were 4.2 (± 0.2), 3.3 (± 0.7), and 1.7 (± 0.9), respectively. The results of univariable linear regression analysis indicated that bacterial load in log_10_ is a strong predictor of reduction in cell counts in log_10_ (P < 0.0001, R^2^ = 0.72), such that: reduction in cell counts = 5.5–1.2 × bacterial load (Fig 7)

### Changes in surface properties

There were no apparent changes in material structure, color, and roughness of any of the five material surfaces.

## Discussion

The first major finding of this study was that aqueous O_3_ at concentration of 4 ppm is sufficient to reduce manure-based pathogens to a safe level (> 5-log) on smooth surfaces within 2 minutes exposure. The second major finding was that a single exposure to either aqueous or gaseous O_3_ is not an adequate means of controlling manure-based pathogens under heavy bacterial load in complex environments. The third major finding was that the 4 minutes exposure is the optimal time of exposure for aqueous O_3_. Finally, it is important to note that surface bacterial load does not alter bacterial killing dynamics of O_3_ on smooth surfaces, but has a significant impact on bacterial killing dynamics of O_3_ on complex surfaces.

To our knowledge, this is the first study that has described the killing capacity of aqueous and gaseous O_3_ at different operational conditions to effectively inactivate the manure-based pathogens to a safe level on different surfaces that can be found in livestock and food production operations. The results of this study demonstrated on smooth surfaces a significant impact of O_3_ concentration on RF, where increasing aqueous O_3_ concentration from 2 to 4 ppm was sufficient to decrease the MBP below detectable limits within 2 minutes. Interestingly, the presence of organic matter and the physiological differences among bacteria such as the thickness, density, and composition of bacteria cell wall and inner membranes had no influence on bacterial killing dynamics of aqueous O_3_ at 4 ppm on smooth surfaces. Moreover, it has been reported that E. coli develops mechanisms such as SoxR and OxyR to protect against the lethal effect of O_3_. These mechanisms activate various genes like *soxS* and *sod* that protect E. coli cell against the oxidative stress of O_3_ through DNA repair or removal of the radicals [29]. However, these protection mechanisms didn’t’ seem to hold up for long at this dose of O_3_. Therefore, the safe level of MBP could be achieve with O_3_ levels of 4 ppm or greater on smooth surfaces with at least 2 minutes exposure. These findings are consistent with the results of earlier studies that reported significant impact of O_3_ concentration and rate of mass transfer on RF [30–32].

The increased bacterial reduction on smooth surfaces could be explained by the fact that smooth surfaces enhance the mass transfer of O_3_ resulting in an efficient and faster diffusion of O3 molecules among manure-based microbes and consequently efficient and faster penetration and destruction of microbes [33]. Therefore, the lag time, the period where the O_3_ molecules react with microbes, is reduced significantly with high concentration of O_3_ on smooth surfaces [32]. Moreover, smooth surfaces reduce the surface ozone reactivity, irreversible consumption of O3 when it strikes the surface, to the lowest degree resulting in decrease the physical removal of O_3_ [34]. Furthermore, decrease the adhesion of microbes to such simple surface makes them more vulnerable to O_3_ [35].

The results of RF on intermediate surfaces support the idea that as the surface increases in complexity the killing dynamics of O_3_ are altered. In all cases as the complexity-roughness of the surface increases there is an increased adhesion of microbes and ozone-oxidizable materials on the surface [32]. With increasing adhesion, the microbes and organic matter in the upper layers prevent O_3_ from reaching underneath microbes [36–37]. Additionally, because of the O_3_ flux limitation, adhesion slows diffusion which negatively impacts bacterial killing dynamics [38]. On metal surfaces RF was lower than on plastic as the O_3_ destruction rate increased due to increased surface-ozone reactivity as a result of the easily oxidized iron and manganese that are present [39]. Additionally, iron bacteria have a strong binding affinity for metals, even with galvanic coating, where some of these bacteria provide tolerance to the high zinc concentration environment results in limiting O_3_ contact to microbes [40]. It has been reported that, O_3_ reacts with a nylon surface, increasing its hydrophilic properties which results in an increase the water penetration into internal structures that leads to an increased removal rate of O_3_ [41]. While the reasons and degree vary between these more complex surfaces, a higher O3 concentration is required in order to achieve reduction in manure-based pathogens below detectable limits.

The rate of bacterial reduction on rubber surfaces is decreased, compared to plastic, metal, and nylon, suggesting that the physical and chemical properties of rubber material negatively impact the killing dynamics of O_3_ [36]. Ozone attacks the double bonds in the polymeric chains present in such polypropylene, polyethylene, and polyethylene terephthalate surfaces. As the surface is damaged with O_3_, the underneath bonds will be then exposed and react with O3 creating cracks resulting in excessive surface-ozone reactivity [42–43]. It would be expected that the adhesion dynamics of MBP and organic matter would be complicated and the diffusion O_3_ on rubber surface would be limited. Therefore, increasing the concentration of O_3_ is necessary to achieve a safe level of bacterial reduction. Interestingly, a higher RF was reported after 8 minutes exposure, however the O_3_ is continued to decompose in water with time, which might be explained by the hidden of bacteria in minute cracks created by reaction of O_3_ and rubber surface that were not eluted off the surface during the washing process.

The lowest reduction rate of manure-based pathogens was on wood surfaces and this could be explained by the highest surface-O_3_ reactivity. Wood-based materials are complex heterogenic materials with high molecular weight components, particularly cellulose (40% to 50%); lignin (15% to 35%); hemicellulose (20% to 35%), and solvent-soluble extractives (3% to 10%) such as terpenes, tannins, aromatic and aliphatic acids [44]. In the paper industry, ozone is usually used for delignification of wood products as it is a strong reactant to degrade lignin [45–46]. Moreover, the wood materials releases O_3_-reactive substances (volatile organic compounds) that consume O_3_ before reaching microbes in the irregular pores of wood surface [47]. Ozone also reacts with unsaturated hydrocarbons that are found in wood products resulting in the increase rate of O_3_ breakdown [48]. With result to O_3_ diffusion, O_3_ flux on wood surface is limited due to loss of O_3_ in the irregular porous layer [32].

Our results demonstrated a significant impact of O_3_ concentration on the rate of bacterial reduction. This finding is consistent with the results of other studies investigating the effect of concentration of O_3_ that is dissolved in water on the reduction in MBP [25, 30–31, 49–50]. The minimal bactericidal concentration of O_3_ is still an area of debate, where Hamelin and Chung [29], demonstrated that the minimum bactericidal concentration of O3 is 1 ppm, whilst other studies have recommended values between 3 ppm and 4 ppm [25; 31, 50] and 20 to 30 ppm for gaseous O3 [51, 52]. On the other hand, we didn’t identify significant impact exposure time on RF especially at higher concentrations except rubber. Comparable results were reported in the earlier study, where no significant difference between 5 and 10 minutes exposure at concentration higher than 2.5 ppm were reported [53]. The full killing power of O_3_ occurs mostly during the first 4 minutes of exposure then starts to decompose [54], where aqueous O_3_ concentration reduced to half within 4 to 5 minutes even with high quality water and low concentrations of oxidizable organic material, iron and manganese [49, 55].

Based on the results of this study, gaseous O_3_ cannot be an alternative to aqueous O_3_ in decreasing the manure-based pathogens to a safe level, especially in complex environment. The same results were reported in several previous studies [22–24, 50, 55–57]. This might be attributed to the maximum concentration of gaseous O_3_ used in this study is lower than the minimal bactericidal that has been recommended in earlier studies [51, 52]. Moreover, the times of exposure for gaseous O_3_ used in the present study were short comparing to earlier studies, where there is consensus that increase the concentration of gaseous O_3_ exposure time in hours will increase the rate of bacterial reduction [50, 58]. The results of recent studies supported the earlier findings, where Sharma and Hudson in 2008 used gaseous O_3_ of 25 ppm for 20 minutes reported ≥ 4-log_10_ reduction in gram negative bacteria on plastic surfaces; while, Wani and others [50] reported killing of approximately 6- log_10_ of E. coli on blood agar media when used 50 ppm for 10 minutes. The different bacterial reduction rates for different surfaces reported here support the earlier finding that killing capacity of O_3_ is surface dependent. Gaseous O_3_ of 10 ppm for 10 minutes reduced the bacterial population by 3-log_10_ on blood agar [50], and only 1.4-log_10_ on spinach after 3 days of exposure [58]. Moreover, some bacteria need more time (hours) to be destroyed using gaseous O_3_ [59]. Other potential reason might be related to the density of air. Gaseous O_3_ of 1.0 ppm equates to 2.14 mg O_3_/m^3^_,_ however 1 ppm of aqueous ozone equates to 1 g O_3_/m^3^ water [60]. Therefore, a high concentration of gaseous O_3_ is required for an efficient reduction of a bacterial population.

To our knowledge, this is the first study investigating the effect of bacterial load on killing capacity of O_3_ and the results confirm our hypothesis that at the same concentration increasing the bacterial load decreases the rate of bacterial killing secondary to the consumption of more O_3_ molecules by the bacteria. Therefore, measuring bacterial load can be meaningful predictor of bacterial reduction at a given concentration and can serve as a guide to identify the optimal operational conditions of O_3_ that should be used to achieve a safe level of MBP reduction. This study provides a practical guide for optimal operational conditions of aqueous and gaseous O_3_ for controlling MBP in dairy operations. However, there were some limitations in this study. First, our study was conducted in a controlled environment and additional studies are indicated to determine the external validity of the results.

## Conclusions

Aqueous O_3_ of 4 ppm may provide a sufficient method to reduce MBP to a safe level (> 5-log_10_) on plastic and metal surfaces, while aqueous and gaseous O_3_ alone may not an adequate means of controlling MBP under heavy bacterial load in complex environments. We also support the recommendations of using 4 minutes as an optimal time of exposure for aqueous O_3_. The bacterial load does not alter bacterial killing dynamics of O_3_ on smooth surfaces, but has a significant impact on bacterial killing dynamics of O_3_ on complex surfaces in this study.

## Supporting information

S1 Fig Mean ± SD log_10_ reduction in MBP cell counts on the surface of substrate. (A) Plastic, (B) metal, (C) nylon, (D) rubber, and (E) wood substrates contaminated with dairy cattle manure and treated with aqueous O_3_ of 2, 4, and 9 ppm for 2, 4, and 8 minutes exposure. The horizontal dashed line indicates 100 killing percentage (mean cell counts of the control groups at the same concentration and time point). Concentrations at the same time point with different capital letters differ significantly (*P* < 0.05). Time points with different small letters within one concentration differ significantly (P < 0.05).
(TIF)

S2 Fig Boxplot of log_10_ reduction in MBP cell counts on the surface of substrate. (A) Plastic, (B) metal, (C) nylon, (D) rubber, and (E) wood materials contaminated with dairy cattle manure and treated with gaseous O3 of 1 and 9 ppm for 2, 4, 8 minutes exposure. The horizontal dashed line indicates 100 killing percentage (mean cell counts of the control groups). The blue diamond indicates mean. *Values differ significantly between O_3_ concentrations at the same time point (P < 0.05).
(TIF)
