# Supplementary figures and images for: The microbial killing capacity of aqueous and gaseous ozone on different surfaces contaminated with dairy cattle manure
Source: PLoS One. 2018 May 14;13(5):e0196555. doi: 10.1371/journal.pone.0196555 (PMC5951574; doi:10.1371/journal.pone.0196555)

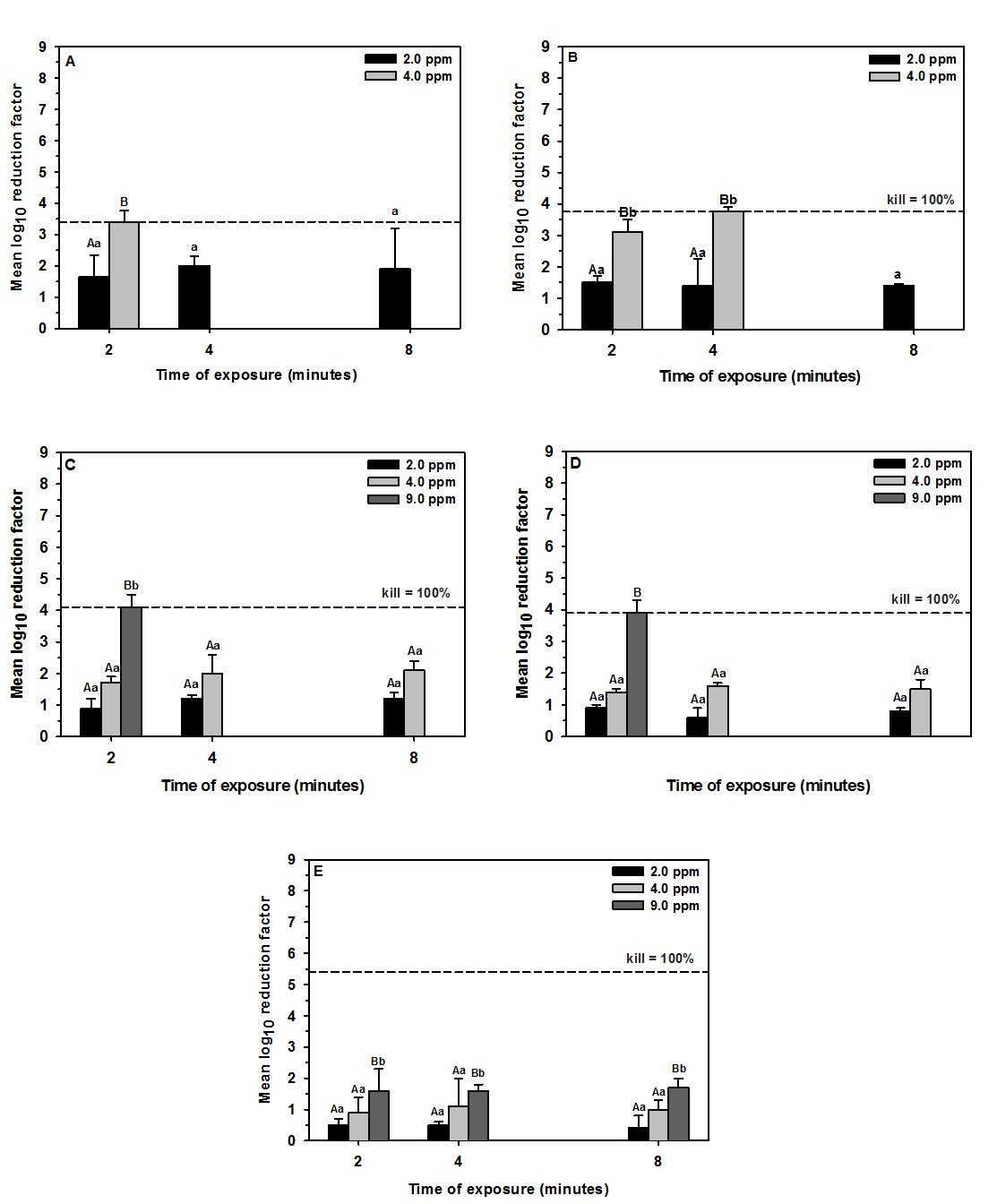

Supplement: S1 Fig — (A) Plastic, (B) metal, (C) nylon, (D) rubber, and (E) wood substrates contaminated with dairy cattle manure and treated with aqueous O3 of 2, 4, and 9 ppm for 2, 4, and 8 minutes exposure. The horizontal dashed line indicates 100 killing percentage (mean cell counts of the control groups at the same concentration and time point). Concentrations at the same time point with different capital letters differ significantly (P < 0.05). Time points with different small letters within one concentration differ significantly (P < 0.05). (TIF) [file pone.0196555.s001.TIF]

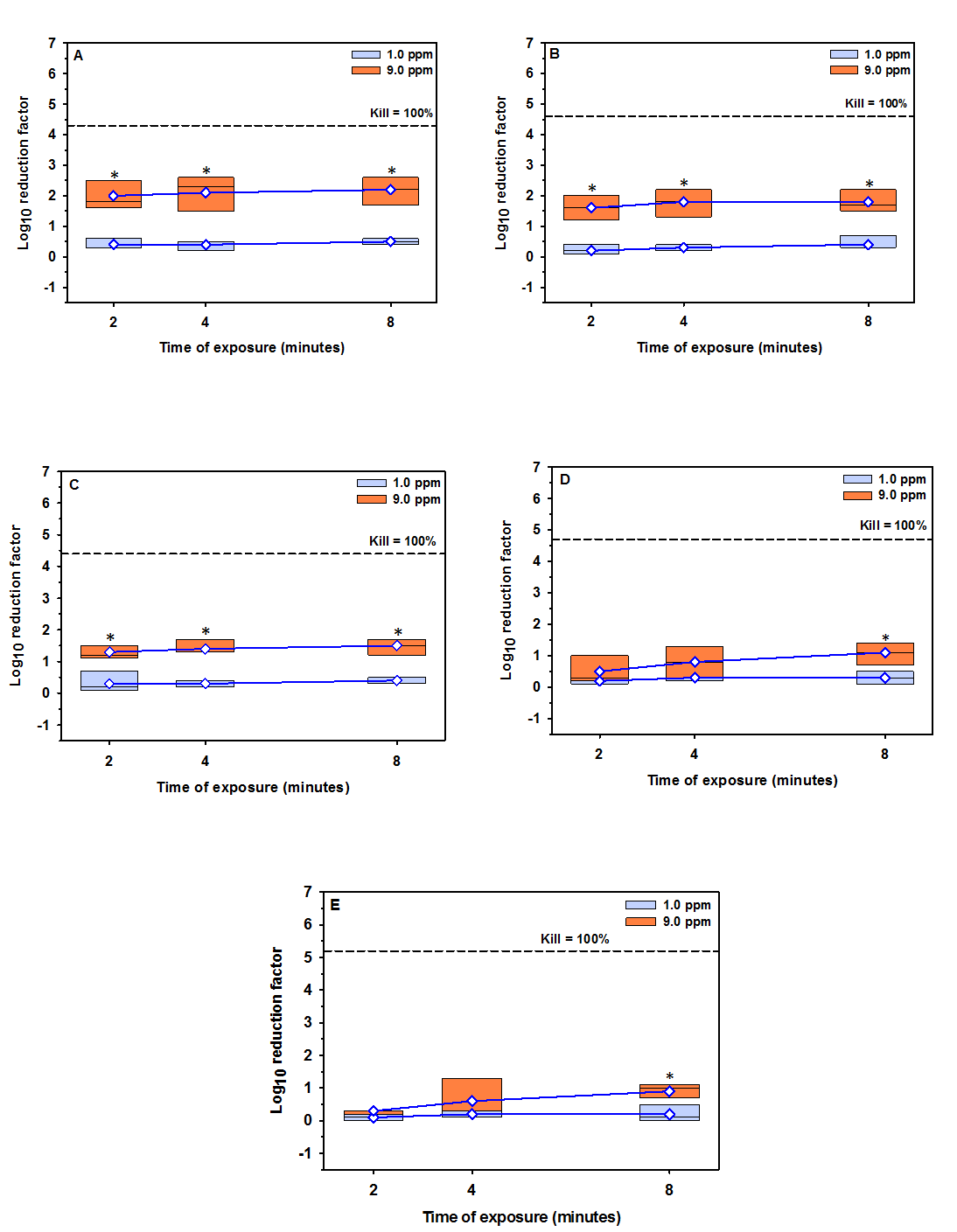

Supplement: S2 Fig — (A) Plastic, (B) metal, (C) nylon, (D) rubber, and (E) wood materials contaminated with dairy cattle manure and treated with gaseous O3 of 1 and 9 ppm for 2, 4, 8 minutes exposure. The horizontal dashed line indicates 100 killing percentage (mean cell counts of the control groups). The blue diamond indicates mean. *Values differ significantly between O3 concentrations at the same time point (P < 0.05). (TIF) [file pone.0196555.s002.TIF]
